# Effect of Cleansing Agents on the Color Stability of Stained Clear Aligners: An In Vitro Study

**DOI:** 10.7759/cureus.98327

**Published:** 2025-12-02

**Authors:** Ernesta Jancauskaite, Migle Dainauskaite, Agne Baliutaviciute, Giedre Uze, Rasa Baniene, Kristina Lopatiene, Arunas Vasiliauskas

**Affiliations:** 1 College of Medicine, Lithuanian University of Health Sciences, Kaunas, LTU; 2 Dentistry, Lithuanian University of Health Sciences, Kaunas, LTU; 3 Prosthodontics, Lithuanian University of Health Sciences, Kaunas, LTU; 4 Biochemistry, Lithuanian University of Health Sciences, Kaunas, LTU; 5 Orthodontics, Lithuanian University of Health Sciences, Kaunas, LTU

**Keywords:** clear aligners appliance, color, orthodontics, staining and labeling, vita easy shade

## Abstract

Aim

This study aims to assess the discoloration of clear orthodontic aligners caused by commonly consumed beverages and to compare the effectiveness of various cleaning agents in stain removal.

Materials and methods

A total of 100 clear orthodontic aligners were immersed for seven days in four commonly consumed beverages, specifically black coffee, black coffee with milk, kombucha, and a green smoothie, as well as in distilled water, which served as the control solution. Following this immersion period, 20 aligners from each beverage group were cleaned using four different cleansing agents: Bluem Aligner Foam, Curaprox BDC 100 Denture Gel Daily, IsoDent Ortho Cleaner, and 3% hydrogen peroxide. The VITA Easy-Shade compact spectrophotometer used the CIELAB (Commission Internationale de L'Eclairage L*a*b* colour system) scheme to evaluate the color change of the aligners at four intervals: T0 (before immersion), T1 (after 24 hours), T2 (after 48 hours), T3 (after seven days), and T4 (after cleaning with cleansing agents). The color values were converted into National Bureau of Standards (NBS) units to quantify the degree of color change. Statistical analysis was performed using IBM SPSS Statistics for Windows, Version 29 (Released 2022; IBM Corp., Armonk, New York, United States), employing non-parametric methods to assess differences between groups and across time intervals.

Results

A significant color change in aligners was observed after immersion in the stated solutions (p < 0.001). Among the staining agents, black coffee induced the highest and most consistent colour change at T0-T3 ΔE = 8.867 (8.553-9.22), followed by coffee with milk ΔE = 3.325 (3.259-3.544), kombucha ΔE = 2.45 (2.272-3.094), and green smoothie ΔE = 1.581 (1.463-1.723) (p < 0.001). Intergroup and intragroup comparisons of cleansing agents revealed no significant differences among groups.

Conclusion

The study demonstrated significant differences in the staining potential of various beverages on clear aligners, with black coffee inducing the most pronounced discoloration. All aligners exhibited progressive color changes over time. The cleansing agents tested showed limited efficacy, with no statistically significant ability to reverse the discoloration.

## Introduction

Clear aligner therapy (CAT) has become a highly popular method in orthodontic treatment due to its aesthetic and convenient approach. Aligners are more comfortable, less noticeable, and offer better aesthetics compared to traditional fixed braces [1,2]. A recent survey revealed that 93.13% of orthodontists in Australia offer CAT as part of their treatment options [3]. In the United Kingdom and the Republic of Ireland, 77.3% of surveyed orthodontists reported incorporating CAT into their clinical practice [4]. In addition, 65% of respondents from the United States and Canada indicated that they utilize CAT in their orthodontic treatments [5].

However, the effectiveness of CAT relies heavily on patient compliance. Research indicates that 36.0% of patients demonstrated full compliance, 38.3% showed fair compliance, and 25.7% exhibited poor compliance [6]. Wearing aligners near-permanently is key, as poor compliance can lead to delays or changes in treatment. Therefore, it is essential to identify daily habits that might prevent patients from wearing their aligners properly.

Clear aligners are favoured by patients for their low visibility and transparency. To ensure optimal visual clarity, aligner materials should possess high light transmittance, ideally transmitting no less than 80% of visible light [7]. During the standard wear period of one to two weeks, the transparency of a clear aligner should remain consistent [8]. Clear aligners’ transparency and colour stability may deteriorate due to coloured beverages, UV light exposure, and specific mouthwashes [9].

In recent years, more attention has been given to appliance care, colour stability, and the effects of staining foods and drinks. Discoloration of aligners is one factor that can influence patients’ wearing habits. Aligners tend to discolour when in contact with staining foods [10]. Therefore, it is recommended to remove the appliance while eating or drinking [11]. However, because of their busy lifestyles, patients often avoid removing their aligners while drinking for reasons such as embarrassment about taking them out in public, feeling unhygienic when reinserting them in shared spaces, and fear of losing or damaging the aligners when they are not being worn.

Research shows that aesthetic reasons are the leading motivation for patients to seek treatment; therefore, we believe that the appearance of aligners plays an important role, especially since clear aligners have revolutionized aesthetic orthodontic treatment [12,13]. Clinical knowledge about the color stability of aligners is crucial when providing wear recommendations to patients. By educating patients about certain staining foods and beverages, clinicians can help prevent aligner discoloration and reduce compliance issues related to aesthetic concerns.

This in-vitro study aims to evaluate the staining effects of various commonly consumed beverages on clear orthodontic aligners and to compare the cleaning efficacy of different cleansing solutions after staining, with color changes assessed via spectrophotometric analysis. It is hypothesized that commonly consumed beverages will cause measurable and statistically significant discoloration of clear orthodontic aligners, and that aligner cleansers will be effective in reducing this discoloration.

## Materials and methods

Aligner material collection

The study was conducted at the Lithuanian University of Health Sciences, Kaunas, Lithuania. Clear aligners from the Ordoline brand (UAB Ordoline, Vilnius, Lithuania) were used. A total of 100 unused clear aligners (50 maxillary and 50 mandibular) were included. The sample size of 100 specimens was selected to ensure adequate statistical power for detecting moderate differences in color stability (ΔE) between conditions. Assuming a conventional α of 0.05 and a medium effect size (Cohen’s d ≈ 0.5), a total sample of 100 provides approximately 80% power for typical comparisons in in-vitro color-stability studies, which is consistent with recommended guidelines for laboratory research. The samples were divided into five groups of 20 specimens each.

In this study, four commonly consumed beverages were chosen for the immersion of the samples: black coffee (NESCAFÉ® Classic; Nestlé S.A., Vevey, Switzerland), black coffee with milk (NESCAFÉ® Classic with UAT FARM MILK, 3.2% fat), kombucha (SUN365), and green smoothie (SUN365). Additionally, distilled water was used as the control group.

Preparation

**Figure 1:**
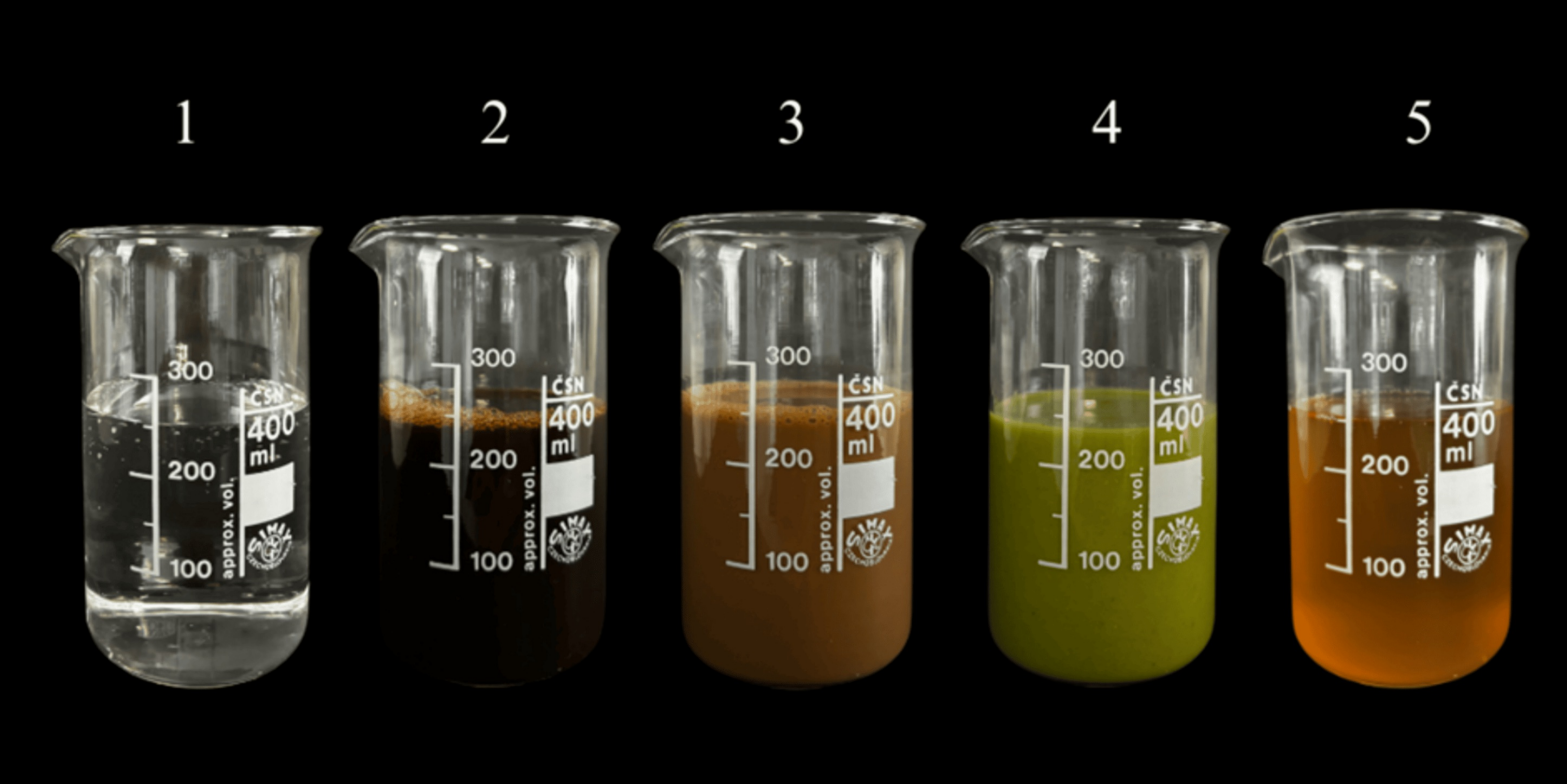
Preparation of solutions. Solutions: (1) distilled water, (2) black coffee, (3) coffee with milk, (4) green smoothie, and (5) kombucha.

All test beverages and experimental samples were prepared using the same protocol, ensuring consistency across all experimental conditions. Black coffee was prepared by dissolving 17,65 g of coffee grains in 300 mL of boiling distilled water (ratio 1:17), the second solution was prepared by mixing 17,65 g of coffee grains with 200 mL of boiling distilled water and 100 mL of milk (ratio 1:17). Both solutions were cooled to room temperature and filtered using a filter paper to remove any residues. The other two solutions, kombucha and green smoothie, were used directly from the bottle, without further preparation. Each solution was poured into separate containers (Figure 1) and placed into an incubator to achieve a temperature of 37°C. Tooth-shaped models of the left maxillary (tooth no. 21) and right mandibular (tooth no. 41) central incisor crowns were made using packable composite, shade A2 (Charisma®, Kulzer GmbH, Hanau, Germany). The central incisors were selected on the basis of their position within the aesthetic zone and their clinical relevance as the area most susceptible to visible aligner discoloration. For color assessment, the models were placed within the clear aligners during color change measurements. All colour measurements were performed at the center of the palatal surface of the upper left central incisor (tooth 21) and the middle of the lingual surface of the lower right central incisor (tooth 41). These specific measurement sites were chosen to minimize potential inaccuracies associated with attachments.

Procedure

Before immersion in the test solutions, each tooth model was first placed in its respective aligner for baseline color evaluation (T0) using a spectrophotometer. The tooth model was then removed, and the aligners were immersed in the different solutions.

Following 24 hours of immersion, the aligners were rinsed with distilled water and dried with tissue paper. The tooth model was then repositioned in the aligner for the second color evaluation (T1) using the spectrophotometer. The aligners were subsequently re-immersed in the same solutions, and additional measurements were obtained at 48 hours (T2) and at seven days (T3).

At the end of the seven-day period (T4), 20 clear aligners from each beverage group were cleaned using four different cleansing agents: Blue®m Aligner Foam (Blue®m Oral Care, Netherlands), Curaprox BDC 100 Denture Gel Daily (Curaden AG, Kriens, Switzerland), IsoDent Ortho Cleaner (IsoDent Ltd., Helsinki, Finland), and 3% hydrogen peroxide. The distilled water control group was excluded from this cleaning procedure. In total, 80 aligners were cleaned.

Colour change evaluation

A standard VITA Easyshade compact spectrophotometer (VITA Zahnfabrik, Bad Säckingen, Germany) was used to check the change in colour, which was evaluated at four intervals. T0: before immersing into the solution, T1: 24 hours after being immersed, T2: 48 hours after being immersed in the solution, T3: seven days. All the measurements were done in the same room with a standardized light source, under a neutral grey background. Blinding of the examiners during colour measurements was not implemented.

A single tooth colour evaluation setting mode was selected to obtain CIELAB (Commission Internationale de L'Eclairage L*a*b* colour system) data. The colour parameter L* represents the lightness (+ is lighter, - is darker), a* is the red/green coordinate (+ is redder, - is greener), and b* is the yellow/blue coordinate (+ is yellower, - is bluer). Colour changes of the clear aligner samples were calculated using the following formula: ΔE=√(ΔL)2+(Δa)2+(Δb)2. The colour change rating was done with the help of the National Bureau of Standards System (NBS). The ΔE value was converted into NBS units for clinical relevance with the formula NBS = ΔE × 0.92 (Table 1).

**Table 1:** Critical levels of colour changes in line with NBS system. ΔE: color difference; NBS: National Bureau of Standards ratings

ΔE	NBS criteria
0.0-0.5	Trace: remarkably slight alteration
0.5-1.5	Slight: slight alteration
1.5-3.0	Noticeable: observable alteration
3.0-6.0	Appreciable: apparent alteration
6.0-12.0	Much: remarkably apparent alteration
12.0 and more	Very much: alteration to another colour

Cleansing agent’s comparison

After the immersion and color evaluation phases, a subsequent experiment was carried out to compare the cleaning effectiveness of four cleansing agents on stained clear aligners: Blue®m Aligner Foam, Curaprox BDC 100 Denture Gel Daily, IsoDent Ortho Cleaner, and 3% hydrogen peroxide. Twenty clear aligners from each beverage group - black coffee, black coffee with milk, kombucha, and green smoothie - were cleaned using different cleansing solutions. The distilled water group was not included in this assessment. A total of 80 specimens were treated with the cleansing agents.

In the first group, Blue®m Aligner Foam was evenly applied to the stained aligners and left for five minutes. For the second group, two to three drops of Curaprox BDC 100 Denture Gel Daily were applied and left for the same amount of time. In the third group, 15 mL of IsoDent Ortho solution was mixed with 200 mL of warm distilled water, and the aligners were fully submerged for 20 minutes. The final group was treated with a mixture of 100 mL of hydrogen peroxide and 100 mL of warm distilled water, with aligners submerged for 20 minutes. After treatment, all aligners were gently brushed with a soft toothbrush (Curaprox® CS 5460) for two minutes, rinsed with distilled water, and dried with a paper towel. Colour changes (T4) were then evaluated following the same protocol.

Statistical analysis

The obtained data were statistically analysed using IBM SPSS Statistics for Windows, Version 29 (Released 2022; IBM Corp., Armonk, New York, United States). The Shapiro-Wilk test indicated that most data were not normally distributed (p < 0.05); therefore, non-parametric statistical methods were applied. After calculating ΔE and NBS values using the formulas described above, descriptive statistics were obtained for each group. The Kruskal-Wallis test was used to compare colour change values among different staining agents, and when significant differences were observed, pairwise Mann-Whitney U tests with Bonferroni correction were conducted to identify specific intergroup differences. For intragroup comparisons across multiple time points, the Friedman test was applied to assess the progression of colour changes within each solution. Pairwise Wilcoxon signed-rank tests were subsequently performed for comparisons between specific time intervals.

Cleaning efficacy was evaluated using the Friedman test for intragroup analysis, followed by Wilcoxon signed-rank tests comparing colour change before (T0-T3) and after cleaning (T0-T4) within each staining group. Differences among cleansing agents at (T3-T4) were assessed using the Kruskal-Wallis test, followed by a pairwise Mann-Whitney U test. A p-value of < 0.05 was considered statistically significant.

## Results

**Figure 2:**
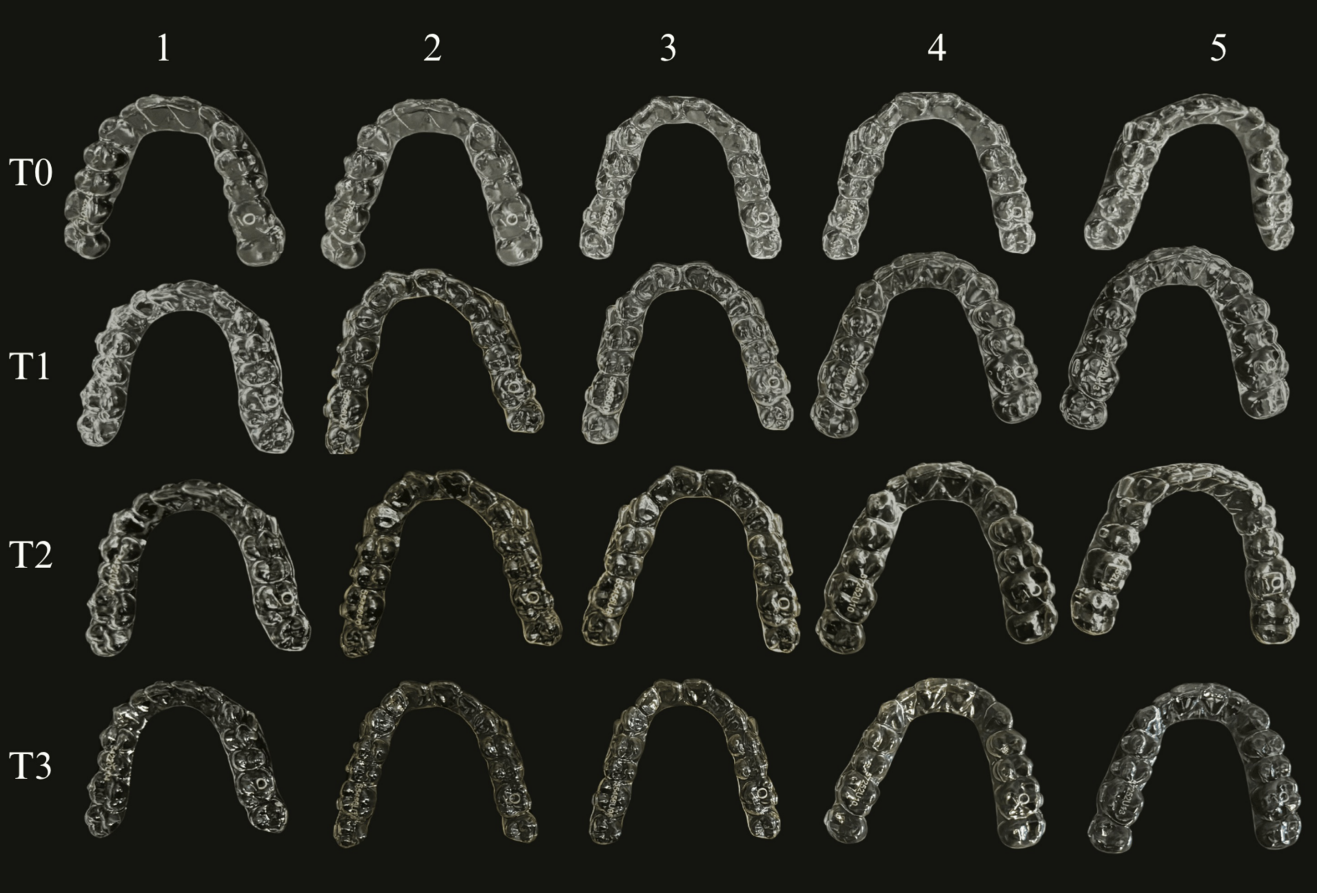
Photographs of the aligners before and after immersion. Solutions: (1) distilled water, (2) black coffee, (3) coffee with milk, (4) green smoothie, and (5) kombucha. Time intervals: (T0) before immersing into the solutions, (T1) 24 hours after being immersed, (T2) 48 hours after being immersed in the solution, and (T3) seven days.

Photographs of the aligners before and after immersion (24 hours, 48 hours, and seven days) in distilled water, black coffee, coffee with milk, green smoothie, and kombucha are presented in Figure 2. Visual inspection confirmed that all aligners were transparent prior to immersion in the solutions.

Intergroup comparisons (Kruskal-Wallis test) demonstrated statistically significant differences in colour stability among all tested solutions (p < 0.001). The control group (distilled water) showed a negligible colour change over all time intervals, confirming its suitability as a baseline. Among the staining agents, black coffee induced the highest and most consistent colour change at T0-T3 ΔE = 8.867 (8.553-9.22), (p < 0.001), followed by coffee with milk ΔE = 3.325 (3.259-3.544), (p < 0.001), and kombucha ΔE = 2.45 (2.272-3.094), (p < 0.001). Green smoothie also resulted in measurable discolouration, though to a lesser extent, ΔE = 1.581 (1.463-1.723), (p < 0.001) (Table 2).

**Table 2:** Colour stability changes in different solutions over time (ΔE comparison). Solutions: (1) distilled water, (2) black coffee, (3) coffee with milk, (4) green smoothie, and (5) kombucha. Time intervals: (T0) before immersing into the solutions, (T1) 24 hours after being immersed, (T2) 48 hours after being immersed in the solution, and (T3) seven days.

Time intervals	Solutions	N	Mean	Standard deviation	Median	Q1	Q3	P-value
									T0-T1	1	20	0.109	0.087	0.1	0	0.143	<0.001
										2	20	4.066	0.923	3.852	3.45	4.657	
3	20	0.982	0.183	0.949	0.877	1.082											
4	20	0.665	0.13	0.608	0.608	0.714											
5	20	0.568	0.083	0.592	0.51	0.592											
T0-T2	1	20	0.117	0.058	0.1	0.1	0.173	<0.001
	2	20	5.834	0.696	5.62	5.389	6.304	
	3	20	2.062	0.294	2.195	1.879	2.256	
	4	20	0.995	0.135	0.995	0.906	1.039	
	5	20	1.297	0.383	1.183	1.02	1.269	
T0-T3	1	20	0.131	0.052	0.1	0.1	0.185	<0.001
	2	20	8.843	0.584	8.867	8.553	9.22	
	3	20	3.376	0.151	3.325	3.259	3.544	
	4	20	1.627	0.181	1.581	1.463	1.723	
	5	20	2.591	0.411	2.45	2.272	3.094	
T1-T2	1	20	0.054	0.057	0.05	0	0.1	<0.001
	2	20	2.062	0.299	2.001	1.913	2.245	
	3	20	1.105	0.248	1.116	1.049	1.3	
	4	20	0.35	0.083	0.316	0.3	0.412	
	5	20	0.739	0.366	0.592	0.548	0.678	
T1-T3	1	20	0.806	0.076	0.1	0	0.141	<0.001
	2	20	4.932	0.53	4.808	4.5	5.394	
	3	20	2.405	0.187	2.373	2.272	2.474	
	4	20	0.98	0.192	0.927	0.877	1.025	
	5	20	2.04	0.391	1.909	1.682	2.423	
T2-T3	1	20	0.04	0.05	0	0	0.1	<0.001
	2	20	3.019	0.299	3.125	2.739	3.209	
	3	20	1.33	0.266	1.263	1.086	1.526	
	4	20	0.65	0.219	0.64	0.469	0.7	
	5	20	1.313	0.256	1.234	1.095	1.407	

Pairwise Mann-Whitney post hoc analysis revealed consistent and highly significant differences between distilled water and all staining solutions across all time intervals (p < 0.001). Comparisons among the staining agents showed mixed results: while most pairings were significantly different (p < 0.05), in one interval, no difference was detected (coffee with milk vs. kombucha at T2-T3, p = 1.000) (Table 3).

**Table 3:** Post hoc Mann-Whitney test results for intergroup comparisons of staining solutions at different time intervals. Solutions: (1) distilled water, (2) black coffee, (3) coffee with milk, (4) green smoothie, and (5) kombucha. Time intervals: (T0) before immersing into the solutions, (T1) 24 hours after being immersed, (T2) 48 hours after being immersed in the solution, and (T3) seven days.

Post hoc analysis - Mann-Whitney test						
							T0-T1	Group	1	2	3	4	5
1	-	p < 0.001	p < 0.001	p < 0.001	p < 0.001		
2	U = 0	-	p < 0.001	p < 0.001	p < 0.001		
3	U = 0	U = 0	-	p < 0.001	p < 0.001		
4	U = 0	U = 0	U = 24	-	p = 0.01		
5	U = 0	U = 0	U = 0	U = 84	-		
T0-T2	Group	1	2	3	4	5
	1	-	p < 0.001	p < 0.001	p < 0.001	p < 0.001
	2	U = 0	-	p < 0.001	p < 0.001	p < 0.001
	3	U = 0	U = 0	-	p < 0.001	p < 0.001
	4	U = 0	U = 0	U = 0	-	p < 0.001
	5	U = 0	U = 0	U = 36	U = 74	-
T0-T3	Group	1	2	3	4	5
	1	-	p < 0.001	p < 0.001	p < 0.001	p < 0.001
	2	U = 0	-	p < 0.001	p < 0.001	p < 0.001
	3	U = 0	U = 0	-	p < 0.001	p < 0.001
	4	U = 0	U = 0	U = 0	-	p < 0.001
	5	U = 0	U = 0	U = 12	U = 0	-
T1-T2	Group	1	2	3	4	5
	1	-	p < 0.001	p < 0.001	p < 0.001	p < 0.001
	2	U = 0	-	p < 0.001	p < 0.001	p < 0.001
	3	U = 0	U = 0	-	p < 0.001	p < 0.001
	4	U = 0	U = 0	U = 6	-	p < 0.001
	5	U = 0	U = 4	U = 74	U = 10	-
T1-T3	Group	1	2	3	4	5
	1	-	p < 0.001	p < 0.001	p < 0.001	p < 0.001
	2	U = 0	-	p < 0.001	p < 0.001	p < 0.001
	3	U = 0	U = 0	-	p < 0.001	p = 0.004
	4	U = 0	U = 0	U = 0	-	p < 0.001
	5	U = 0	U = 0	U = 96	U = 0	-
T2-T3	Group	1	2	3	4	5
	1	-	p < 0.001	p < 0.001	p < 0.001	p < 0.001
	2	U = 0	-	p < 0.001	p < 0.001	p < 0.001
	3	U = 0	U = 0	-	p < 0.001	p = 1.000
	4	U = 0	U = 0	U = 16	-	p < 0.001
	5	U = 0	U = 0	U = 200	U = 20	-

When converted to NBS ratings, intergroup comparisons again revealed statistically significant differences (Kruskal-Wallis, p < 0.001). According to the NBS classification, distilled water produced no perceptible colour change (T0-T3). Black coffee resulted in the highest discolouration, ranging from appreciable to much change. Coffee with milk caused moderate staining (slight to appreciable), kombucha induced noticeable changes, while the green smoothie remained within the slight category. This pattern indicates a clear gradient in staining potential: black coffee > coffee with milk > kombucha > green smoothie > distilled water (Table 4).

**Table 4:** NBS-based evaluation of colour changes in different staining solutions over time. Solutions: (1) distilled water, (2) black coffee, (3) coffee with milk, (4) green smoothie, and (5) kombucha. Time intervals: (T0) before immersing into the solutions, (T1) 24 hours after being immersed, (T2) 48 hours after being immersed in the solution, and (T3) seven days. NBS: National Bureau of Standards ratings

Time intervals	Solutions	N	Mean	Standard deviation	Median	Q1	Q3	Description of color change		P-value
											T0-T1	1	20	0.1	0.08	0.092	0	0.152	No colour change		<0.001
												2	20	3.74	0.849	3.544	3.174	4.285	Appreciable		
3	20	0.904	0.169	0.873	0.807	0.995	Slight														
4	20	0.612	0.12	0.56	0.559	0.657	Trace														
5	20	0.522	0.076	0.544	0.469	0.544	Slight														
T0-T2	1	20	0.108	0.054	0.092	0.013	0.159	No colour change		<0.001
	2	20	5.367	0.64	5.17	4.958	5.78	Appreciable		
	3	20	1.897	0.271	2.02	1.729	2.076	Noticeable		
	4	20	0.915	0.124	0.915	0.833	0.956	Slight		
	5	20	1.193	0.353	1.089	0.938	1.167	Slight		
T0-T3	1	20	0.113	0.048	0.096	0.023	0.161	No colour change		<0.001
	2	20	8.136	0.537	8.158	7.869	8.482	Much		
	3	20	3.106	0.139	3.059	2.998	3.26	Appreciable		
	4	20	1.497	0.167	1.455	1.346	1.585	Slight		
	5	20	2.384	0.378	2.254	2.09	2.846	Noticeable		
T1-T2	1	20	0.05	0.052	0.046	0	0.092	No colour change		<0.001
	2	20	1.897	0.275	1.841	1.76	2.065	Noticeable		
	3	20	1.017	0.228	1.026	0.965	1.196	Slight		
	4	20	0.322	0.076	0.291	0.276	0.379	Trace		
	5	20	0.68	0.337	0.544	0.504	0.624	Slight		
T1-T3	1	20	0.74	0.07	0.092	0	0.13	No colour change		<0.001
	2	20	4.538	0.488	4.424	4.14	4.962	Appreciable		
	3	20	2.213	0.172	2.183	2.09	2.276	Noticeable		
	4	20	0.901	0.177	0.853	0.807	0.943	Slight		
	5	20	1.876	0.36	1.756	1.548	2.229	Noticeable		
T2-T3	1	20	0.037	0.046	0	0	0.092	No colour change		<0.001
	2	20	2.777	0.275	2.875	2.52	2.953	Noticeable		
	3	20	1.223	0.245	1.162	0.999	1.404	Slight		
	4	20	0.598	0.201	0.589	0.432	0.644	Slight		
	5	20	1.208	0.235	1.135	1.008	1.295	Slight		

Within-group comparisons over time (Friedman test) indicated significant colour changes in all staining solutions (p < 0.05), whereas distilled water remained stable. Post hoc Wilcoxon analysis confirmed progressive discolouration between early (24 hours, 48 hours) and late (seven-day) intervals across all staining agents (p < 0.001) (Table 5).

**Table 5:** Intragroup pairwise comparisons of colour stability across time intervals (Wilcoxon signed-rank test). Solutions: (1) distilled water, (2) black coffee, (3) coffee with milk, (4) green smoothie, and (5) kombucha. Time intervals: (T0) before immersing into the solutions, (T1) 24 hours after being immersed, (T2) 48 hours after being immersed in the solution, and (T3) seven days.

Solution	Comparison	N	T+	T-	Z	P-value
1	T0-T1 vs T0-T2	20	21	34	│0.668│	1
	T0-T1 vs T0-T3	20	7	48	│2.107│	0.99
	T0-T2 vs T0-T3	20	0	36	│2.558│	0.063
2	T0-T1 vs T0-T2	20	0	210	│3.923│	<0.001
	T0-T1 vs T0-T3	20	0	210	│3.923│	<0.001
	T0-T2 vs T0-T3	20	0	210	│3.923│	<0.001
3	T0-T1 vs T0-T2	20	0	210	│3.923│	<0.001
	T0-T1 vs T0-T3	20	0	210	│3.923│	<0.001
	T0-T2 vs T0-T3	20	0	210	│3.923│	<0.001
4	T0-T1 vs T0-T2	20	0	210	│3.923│	<0.001
	T0-T1 vs T0-T3	20	0	210	│3.923│	<0.001
	T0-T2 vs T0-T3	20	0	210	│3.923│	<0.001
5	T0-T1 vs T0-T2	20	0	210	│3.926│	<0.001
	T0-T1 vs T0-T3	20	0	210	│3.923│	<0.001
	T0-T2 vs T0-T3	20	0	210	│3.923│	<0.001

**Figure 3:**
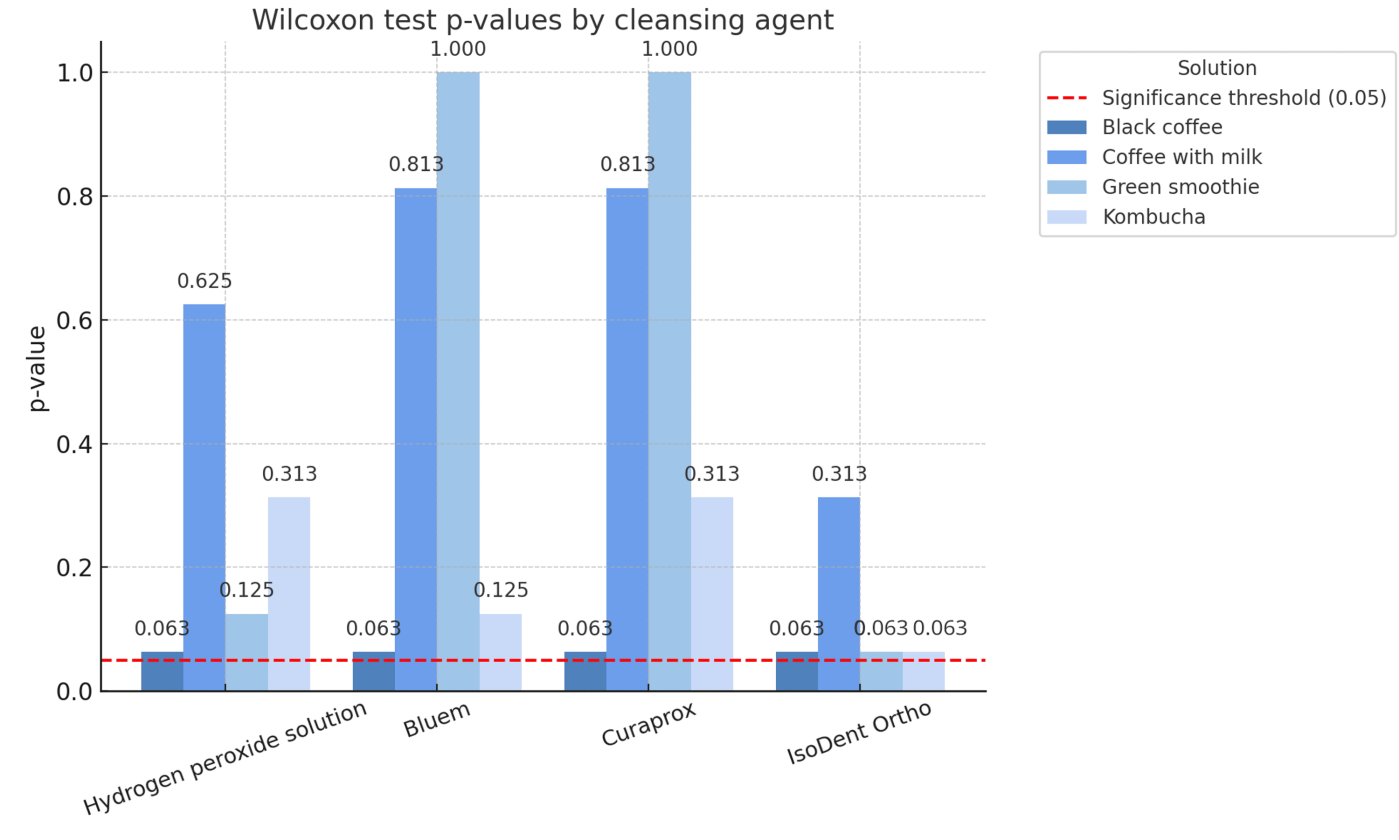
Wilcoxon signed-rank test p-values for intragroup comparisons (before vs. after cleaning) by cleansing agent.

The effectiveness of cleansing agents was further evaluated. The Friedman test demonstrated overall statistically significant differences across solution groups (p < 0.05). In contrast, pairwise Wilcoxon tests did not confirm significant differences for any individual agent (all p > 0.05). Borderline values were observed for black coffee across all agents (p = 0.063) and for IsoDent Ortho Cleaner in the green smoothie and kombucha groups (p = 0.063), whereas no effects were detected for coffee with milk (p ≥ 0.313) (Figure 3).

**Figure 4:**
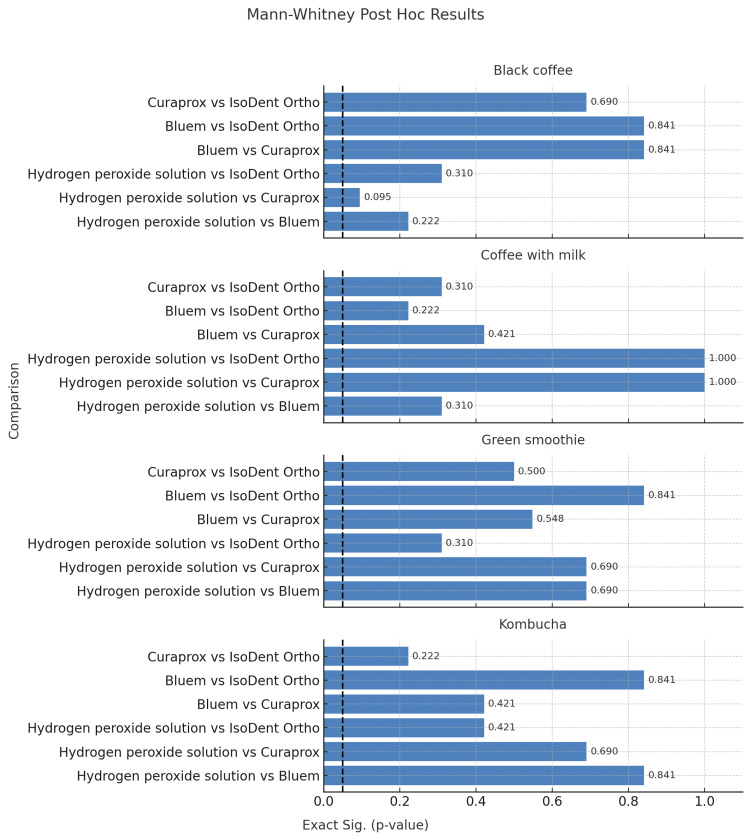
Pairwise Mann-Whitney post hoc comparisons between cleansing agents.

Intergroup comparisons of cleansing agents using the Kruskal-Wallis test revealed no significant differences among groups (p > 0.05). Subsequent pairwise Mann-Whitney post hoc tests likewise showed no statistically significant differences between cleansing agents for any staining solution (all p > 0.05). The lowest p-value was observed in the black coffee group (hydrogen peroxide solution vs. Curaprox, p = 0.095), but this did not reach statistical significance (Figure 4).

## Discussion

Numerous studies have examined the mechanical, viscoelastic, thermal, and chemical properties of clear aligner materials [14-18], but despite their aesthetic qualities, the visual stability of these materials has received little attention. The current in vitro study was conducted to examine the colour stability of aligner materials when exposed to common beverages. Based on previous research findings and practical experience, the following beverages were selected for the study: coffee, coffee with milk, kombucha (fermented tea beverage), and green smoothie. Distilled water was used as the control solution.

Among all groups in our study, the black coffee group exhibited the most significant discoloration (ΔE = 8.867 between T0 and T3, p < 0.001), with the highest NBS-rated change classified as appreciable to much change. These results are supported by similar studies. In a study by Šimunović et al. [19], coffee caused the highest colour change (mean = 15.156). Lira et al. found that coffee caused significant colour changes after 24 hours (∆E = 11.57 ± 0.69) and further changes after 48 hours (∆E = 13.78 ± 1.12) (p < 0.05) [18]. There is limited evidence on the effect of coffee with milk on clear aligner discoloration, so it was included as a new staining solution in this study. We discovered that coffee with milk caused a notable colour change (ΔE = 3.325), ranking second after black coffee among the tested staining agents, and produced moderate staining from slight to noticeable, as classified by the NBS system. Various studies have found coffee to be a leading factor in discolouring orthodontic appliances and materials, including ceramic brackets, adhesives, and aligners [20]. Coffee discoloration occurs via adsorption and absorption by two materials, likely due to polymer compatibility with coffee’s yellow pigments [21]. Although these results provide useful comparative data, they should be interpreted in light of the in-vitro design, which does not fully replicate clinical exposure conditions such as saliva, intermittent consumption, or temperature changes.

To our knowledge, this is the first study to examine the effect of kombucha on clear aligners. In recent years, more people have become interested in alternative health practices, and kombucha has become a popular drink known for its health benefits, with its market growing quickly [22,23]. Our results showed the total color change of ΔE = 2.450, which corresponds to a "noticeable" level of staining according to the NBS criteria. This value ranks third out of the five tested solutions in terms of staining intensity. Pairwise Mann-Whitney post hoc analysis revealed consistent and highly significant differences (p < 0.001) between distilled water and each of the staining solutions across all time intervals, except for the T2-T3 interval, where no difference was found between the coffee with milk and kombucha solutions (p = 1.000), indicating that they stained similarly during that period.

Due to its high content of natural pigments from leafy greens and fruits, the green smoothie was tested for its effect on aligner colour stability. The green smoothie caused slight but measurable discoloration (ΔE = 1.581), resulting in the least staining among the tested beverages except for distilled water. No similar studies investigating clear aligners and green smoothies were found; however, Veček et al. [24] conducted in vitro research on various dental restorative materials proved that green smoothies caused three to eight times higher ΔE values compared to the control group, indicating significant staining.

Typically, aligners are made from resin-based polymers [25]. Amorphous thermoplastic polymers are highly translucent, making them preferable for clear aligner materials over crystalline polymers, which tend to be opaque and less aesthetic. Polymers such as polyurethane (PU), polyester, polyvinyl chloride, polysulfone, and polycarbonate possess optical properties that make them well-suited for commercial aligner production [8]. In the present study, clear aligners made from an ABA three-layer film consisting of copolyester (A) and thermoplastic elastomer (B), with a detachable isolation foil made of polyethylene (PE), were used. This foil had been removed prior to the experiment. Olteanu et al. [26] compared color changes in two orthodontic clear aligner systems, including one that matched the system used in our study. Results suggest that the PRO group (transparent copolyester and a thermoplastic elastomer) had significantly greater color changes (ΔE values) after immersion in cola (p = 0.025), coffee (p = 0.005), and red wine (p = 0.041) over 14 days compared to the TAG group (PE terephthalate glycol).

Moreover, four different agents were evaluated for their cleaning properties. The tested cleansing agents use different active ingredients to remove discoloration from clear aligners. Blue®m Aligner Foam contains sodium carbonate peroxide, which releases active oxygen to oxidize pigmented molecules. Curaprox BDC 100 Denture Gel Daily has sodium laureth sulfate, a surfactant that helps lift stains from the surface. IsoDent Ortho Cleaner, with orthophosphoric and glacial acetic acids, chemically dissolves mineral and biofilm-related discoloration. And, 3% hydrogen peroxide acts as a strong oxidizer, breaking down color compounds. The Friedman test revealed a statistically significant difference among the cleansing solutions (p < 0.05), suggesting that some agents may perform better than others in stain removal. However, pairwise comparisons using the Wilcoxon test did not confirm significant differences between individual agents (all p > 0.05), indicating that no single cleanser showed a consistently superior effect across all beverage groups.

Based on the results of this in vitro study, it is recommended that patients avoid consuming beverages while wearing clear aligners, as this may affect the colour stability of the appliance and lead to aesthetic issues.

Limitations

There are several important limitations in this study that need to be considered when interpreting the results. The immersion protocol provided continuous contact with staining solutions, which may exaggerate discoloration compared to real-life exposure. In addition, the cleaning procedures were standardized and controlled, whereas patient use may vary in duration, concentration, and frequency, influencing clinical outcomes. The use of tooth-colored composite models as substrates does not fully replicate the optical properties of enamel in vivo, and material variability across different aligner brands and manufacturing batches was not addressed. Furthermore, the study did not examine surface roughness changes, biofilm effects, or saliva interaction, all of which may influence staining clinically. Blinding was not implemented during color measurements, which may have introduced potential measurement bias. In addition, the study did not explore how changes in the temperature of the solutions affect colour alteration. Therefore, investigating their impact on colour stability could be considered in future research.

## Conclusions

This in vitro study demonstrated clear differences in the staining potential of various solutions on clear aligners. Black coffee produced the most pronounced discolouration, followed by coffee with milk, kombucha, and green smoothie, with color changes increasing significantly over time for all staining agents. However, given the continuous and intensified exposure inherent to in vitro conditions, the degree of staining observed may be greater than what occurs in typical clinical use, where exposure is intermittent and influenced by saliva, temperature variation, and individual habits.

The cleansing agents showed limited efficacy, with no statistically significant ability to reverse aligner discoloration. It should be noted that while the lack of statistical significance indicates a limited measurable effect under our study conditions, minor visual improvements may still occur clinically but remain undetectable within the applied statistical thresholds.
